# Examining Predictors of Intention to Leave in Home Care and Differences among Types of Providers

**DOI:** 10.1155/2023/4120204

**Published:** 2023-09-13

**Authors:** Amy J. Hallaran, Sarah Jane Jessup

**Affiliations:** ^1^Trent/Fleming School of Nursing, Trent University, 1600 West Bank Drive, Peterborough K9L 0G2, Ontario, Canada; ^2^Trent University, 1600 West Bank Drive, Peterborough, ON K9L 0G2, Canada

## Abstract

The retention and recruitment of care providers are ongoing concerns in healthcare globally. Examining intention to leave (ITL) as a measure of retention, the existing literature has focused on nurses working in hospitals, with less attention paid to other care providers and other areas of practice. The purpose of this study was to gain a better understanding of the unique factors influencing ITL among three categories of care providers in home care: registered practical nurses, registered nurses, and personal support workers. This study assessed and compared predictors of ITL, including organizational commitment, job satisfaction, perceived supervisor support, burnout, role stress, work/family conflict, and community satisfaction. A convenience sample of home care staff working in one agency in a Canadian province was sent an electronic survey by e-mail in 2021. Responses (*n* = 185) underwent data analysis including descriptive statistics, analysis of variance, and multiple linear regression, as well as thematic analysis of two open-ended items. The results of the study indicated that 54% (*n* = 99) of respondents were considering leaving their job, and respondents were dissatisfied with their salary and benefits. Role stress, work-family conflict, and burnout differed significantly between groups. Several themes emerged for strategies to promote employees to stay with the agency, with the overwhelming strategy being higher wages/salary. Themes for why employees stayed with the agency included love for clients and commitment to their care, as well as fondness for the teams within which respondents worked. The findings of the study lead to several important implications and recommendations for the home care sector. Advocating for wage parity among healthcare sectors and other opportunities for compensation for home care workers is necessary. Additional strategies include supportive and innovative approaches for scheduling, teamwork, and working with staff to identify barriers and solutions in home care.

## 1. Introduction

Recruitment and retention of staff is an ongoing and global issue for healthcare agencies, presenting challenges for maintaining the adequate staffing needed to provide quality care. Intention to leave (ITL) is a common measure used to help assess retention in the workforce. In nursing, leaving positions, employers, and sometimes the profession has been a concern researched for decades. This “turnover” is especially problematic in times of nursing and staff shortages. Within home care in Canada, these concerns are heightened by lower rates of pay and the need to increase capacity to support greater provision of care in the community.

The significance of ITL is better documented in nursing than among unregulated care providers, such as personal support workers (PSWs). Within nursing, the concern of pending shortages over the last two decades has been linked to the aging population and the looming retirement of nurses. In 2009, the Canadian Nurses Association (CNA) reported concerns about significant shortages over the next 15 years in Canada [1] which we are currently acutely experiencing. The intention to leave and subsequent turnover are causes of concern for the quality of patient care and work environments. In addition, there are significant financial implications of ITL due to the reorientation of staff, overtime for covering shifts, and increased absenteeism.

Concerns over turnover and retention are also noted in rural communities, which present additional challenges. This is important to consider in the content of ITL in home care, which spans both urban and rural communities in Canada. Physical isolation, fewer resources, and providers requiring a broader knowledge base, as well as personal factors such as finding jobs for a spouse, a sense of belonging, and relationships with rural organizations have all been reported to influence recruitment and retention in rural settings [2].

The study aimed to better understand the unique factors influencing ITL among the different types of care providers in home care, including RNs, PSWs, and registered practical nurses (RPNs), also known as licenced practical nurses across Canada. Although there is significant literature related to ITL among hospital nurses, there are few studies examining ITL in home care. Understanding these predictors will help inform organizational initiatives targeting recruitment and retention strategies as part of health human resource planning. Prior to the COVID-19 global pandemic, home care was experiencing staffing shortages, which have only worsened with the pandemic and heightened the need to better understand the unique factors in home care that attract and retain staff to ensure quality delivery of care. While this study focuses on home care in Canada, the findings have broader implications for understanding and preventing turnover in home care in various healthcare structures. From the literature reviewed, many regions of the world are grappling with the recruitment and retention of healthcare workers, including in home and community care settings. The findings of this study point to both issues and practical measures in home care that could benefit studies of ITL across the globe.

### 1.1. Conceptual Framework

A conceptual framework of ITL [3] guided the study. The framework proposes that job satisfaction, workplace organization, community characteristics and satisfaction, and individual worker characteristics either directly or indirectly predict ITL [3], which subsequently relates to turnover.

### 1.2. Literature Review

The literature reviewed for ITL identified several predictors, the most important of which are discussed below and align with the components of the conceptual framework.

#### 1.2.1. Intention to Leave

The decision to leave a job or organization is usually superseded by one's intention to leave a job. The construct of ITL is recognized as the greatest predictor of turnover behavior, with corresponding features that can be grouped as workplace factors and personal factors. Attributes of the workplace influencing ITL are readily found in the literature and include organization commitment as one of the most commonly reported predictors, along with job satisfaction, work environment, and empowerment.

In Irvine and Evans [4] seminal study on ITL, they reported that ITL is a key predictor of retention. Yet, measurement of ITL is often inconsistent, and different language is used (e.g., ITL, intention to stay, and intention to search). A literature review conducted by Hayes et al. [5] found that the inconsistent usage of terms in related research can lead to a lack of comparability over time. For example, varying applications of the term “turnover” in ITL studies can make it challenging to monitor trends and compare findings. Despite these concerns, variations in measures of behavioural intentions have continued to be used. For example, some studies have included “intention to stay” as opposed to ITL. This is the case in Berta et al. [6], where researchers examined the relationship among workplace factors and outcomes (i.e., intention to stay) of PSWs in Ontario long-term care and community care settings. Berta et al. reported that intent to stay was significantly associated with job satisfaction, work engagement, and organizational commitment. These findings align with similar studies using the measure of ITL in nurses, suggesting that despite such variations, the findings are comparable and applicable to research examining ITL [7].

#### 1.2.2. Job Satisfaction

Similar to ITL, job satisfaction is variously defined in literature. Despite this potential for variation and subjectivity, studies reveal that there are specific aspects of one's job that are consistently reported to be important to job satisfaction, specifically in the healthcare context. Pay [8], work design, and management support [9] are all factors that influence healthcare workers' job satisfaction and, in turn, their ITL and their decision to stay or leave an employer.

Chamberlain et al. [10] studied individual and organizational predicators of PSWs' job satisfaction in long-term care in Western Canada. Job satisfaction was reported to be an important factor in the intention to leave the job, and lower reported job satisfaction was associated with increased reporting of burnout [10]. Similarly, in their literature review examining international research published between 2000 and 2016, Halcomb et al. [11] ascertained that job satisfaction is consistently found to be a predictor of nurses' intentions to leave or stay. However, the researchers concluded that while the relationship between job satisfaction and ITL is well established in scholarship, the contributing factors to job satisfaction (or dissatisfaction) are less well known. They point to the need for more research on such contributing factors to better understand ITL and thus improve retention rates of nurses.

#### 1.2.3. Workplace Organization

Examinations of workplace organization as a factor in ITL predominantly consider workplace organization in acute care settings [5]. Recent studies that do examine ITL and the role of workplace organization in home care point to the specific elements that influence ITL in home care, including increasing pressures due to an aging population and decreasing support due to limited resources and high turnover [12]. Factors that have been found to encourage home care nurses to remain on the job are similar to those noted for hospitals: greater autonomy, flexible scheduling, manageable workloads, sufficient support, and adequate pay [8]. Looking to fill the gaps in evidence regarding the home care work environment and patient outcomes (rates of hospitalization and discharge), Olga et al. [13] studied RNs in home care agencies across three states (California, New Jersey, and Pennsylvania). Characteristics of a “poor” work environment included longer shifts, whereas “better” work environments had more satisfied nurses and fewer reports of burnout. Olga et al. [13] found that home care agencies with good work environments had lower rates of nurse burnout and hospitalization and higher rates of discharge.

#### 1.2.4. Community Characteristics and Satisfaction

One factor considered in relation to home care or community care nurses is community satisfaction. Stewart et al. [3] reported that community satisfaction was a significant factor associated with ITL for rural and remote nurses across Canada. The researchers assessed items such as community friendliness, social and recreational activities, quality of school, safety, ability to stay current in practice, level of anonymity, size of community, and distance from larger centers.

#### 1.2.5. Individual Characteristics

Typically collected through surveys, analyses of demographic characteristics have identified several individual or personal characteristics that predict ITL. These include age, years of experience, level of education, and home-life obligations such as having children or other dependents [3, 5, 14].

Recently, Möckli and colleagues [15] reported that work-family conflict and work stressors were strongly correlated with emotional exhaustion (a component of burnout) in home care workers in Switzerland. Some specific concerns related to home care that Möckli et al. report include time pressure while commuting and the need for flexible scheduling to promote work-family balance. These unique concerns align with previously reported influences on intention to stay among Ontario home care nurses [8].

In summary, there is limited research examining retention or ITL in home care. The majority of ITL studies have focused on RNs working in hospital settings; hence, there is a gap in knowledge to guide decision-making. The originality of this study is that it seeks to examine factors influencing ITL among different types of workers (RNs, RPNs, and PSWs).

## 2. Research Questions

To identify the predictors of ITL in home care, across different categories of care providers, and to understand the relationship between these predictors, the following research questions were investigated:

1. What is the relationship between personal characteristics, community characteristics and satisfaction, job satisfaction, workplace organization, and ITL?
2. Do the predictors of ITL differ between categories of healthcare providers (RN, RPNs, and PSWs)?
3. What do healthcare providers identify as strategies to promote their retention in home care, and do these differ between categories of healthcare providers (RN, RPNs, and PSWs)?

## 3. Materials and Methods

The research design for the study was quantitative and nonexperimental and incorporated a cross-sectional survey. Prior to commencing the study, ethical approval was obtained from the Trent University's Research Ethics Board (File #26700). The study was initiated in partnership with a home care agency. The home care agency provides services across the province of Ontario, Canada's second-largest province and home to approximately 13.5 million people across one million square kilometers [16] in rural, urban, and remote communities. Services provided by the agency include those funded by the provincial government, such as visiting nursing, personal support and homemaking, and end-of-life care, as well as programs within schools and other residential settings. Options for alternative employment vary greatly across the province, with more options available in highly populated areas. An advisory group from the agency, consisting of five leaders with responsibility for home care delivery across the province, contributed to the proposal development, survey development, and recommendations.

Measurement tools used to assess the key constructs of the framework are identified in Table 1. The measures assessed personal factors, workplace/organizational factors, community characteristics and satisfaction, and ITL. In addition to the tools, demographic questions were included, along with an open-ended question asking participants to identify strategies that would encourage them to stay working in home care and with their current employer. In total, the survey asked participants to rate 108 items and 2 open-ended items, for a total of 121 questions.

### 3.1. Sample

A convenience sample was used. A total of 1038 employees from one home care agency in Ontario, including RNs, RPNs, and PSWs were surveyed. The advisory group provided lists of employee emails for the researcher to distribute the electronic survey by e-mail, which occurred from November to December 2021. A total of 225 respondents opened the survey but only 185 respondents were useable for data analysis. The overall response rate was 18%.

### 3.2. Data Analysis

The raw data was only available to the researchers. Descriptive and inferential statistics were completed using SPSS© (version 28). To answer the first two research questions, data analysis included bivariate correlations, multiple linear regression, and one-way and multivariate analysis of variance. The data from the open-ended questions was analyzed in Microsoft excel by the principal investigator and research assistant, both with previous experience completing thematic analysis. Thematic analysis was used to identify themes in the written responses of participants [26]. Initially, analysis was completed separately, then findings were shared by the principal investigator and research assistant, and congruency was found among themes.

## 4. Results

An equal number of RNs and RPNs completed the survey, 46 in each group, and a larger number of PSWs, 77 completed the survey. Most respondents were employed full-time, female, working one job, and associated with the visiting nursing program.  Table 2 provides characteristics of the sample.

### 4.1. Study Variables

Table 3 provides ranges, means, and reliability coefficients of the measures used for the study variables. Cronbach's alpha ranged from 0.84 to 0.97, including subscales. Given the reliability, minimal missing data (less than 5%), and normal distribution of the data, data analysis proceeded.

#### 4.1.1. Intention to Leave

A total of 99 respondents, 54% of sample, indicated they were thinking about leaving their job in a subsequent question (*If you are thinking about leaving your job, are you considering…*). Respondents who indicated they were thinking about leaving their job could select more than one option to specify their intentions. Respondent selections were as follows: leaving home care (*n* = 62), leaving healthcare (*n* = 32), staying in home care but leaving their current employer (*n* = 27), going back to school (*n* = 27), and moving to a new community (*n* = 10). Many respondents selected “other” (*n* = 43) A variety of comments were received but the most frequent was retirement (*n* = 12). In follow-up, respondents were asked the following subsequent question: If you are considering leaving your job, when do you plan to leave?” Most respondents indicated they were unsure or did not know (*n* = 37). Others responded that they planned to leave within 6 months (*n* = 20), planned to leave within the year (*n* = 16), or planned to leave in greater than 2 years (*n* = 16). Finally, some indicated they were leaving immediately or had taken another job (*n* = 7)

### 4.2. Question 1

The first research questions examined the relationship among personal characteristics, community characteristics and satisfaction, job satisfaction, workplace organization, and ITL. As expected, significant correlations were noted among the study (see Table 4). Multiple linear regression was used to test the conceptual framework of ITL for the entire sample. The overall analysis was significant (*F* = 7.264, *p* < 0.001), and the model accounted for 39% of the variance in ITL (measured by turnover intention). Organizational commitment was significantly associated with ITL (standardized *β* = −0.42, *p* < 0.001). None of the other variables entered in the model significantly relate to ITL.

### 4.3. Question 2

The second research question examined if the predictors of ITL differ between categories of healthcare providers (RNs, RPNs, and PSWs). The data met the assumptions for one-way analysis of variance (ANOVA) and multivariate analysis of variance (MANOVA). Initially, MANOVA determined that there were significant differences among the group means, followed by one-way analysis of variance. The results (see Table 5) identified significant differences in means between groups for the measures of role stress, work-family conflict, and burnout. Post hoc analysis was done using the Bonferroni method to understand the differences in these measures among the groups, with both tests identifying the same significant differences among groups. For RNs, role stress was significantly higher than for PSWs. Examining the subscales of role stress (role ambiguity and role overload), role overload was higher in RNs, contributing to the significant difference. For RNs and RPNs, work-family conflict was significantly higher than that reported by PSWs, although not significantly different between RNs and RPNs. For RNs, burnout was significantly higher than reported by PSWs. Finally, satisfaction with several aspects of the job was also rated. These job aspects included salary, vacation, benefits, scheduling, weekends off, amount of responsibility, orientation and onboarding, opportunities for professional development, traveling, and amount of responsibility. Of these aspects, satisfaction with salary was significantly different between role types (*F* = 7.76, *p* < 0.001), with RPNs reporting lower satisfaction than PSWs and RNs, despite all categories reporting low satisfaction with salary.

### 4.4. Question 3

The third research question explored what care providers identify as strategies to promote their retention in home care and whether there are differences between RNs, RPNs, and PSWs. A total of 156 responses were provided for the two open-ended items, and thematic analysis was completed. The themes that emerged, along with descriptions of the strategies and examples offered by respondents, are presented in Table 6. For strategies to keep staff (i.e., promote retention), three themes emerged: compensation, scheduling, and management opportunities. Overall, the themes were the same between RNs, RPNs, and PSWs with the following exceptions: nursing respondents (RPNs and RNs) suggested more training and education, and RPNs suggested safety concerns be addressed by management.

Respondents were also asked why they stayed with the home care agency. Five themes emerged from these responses (see Table 7) and included patients/clients, nature of community care, the “Team,” work practices, and financial need. Overall, the themes were the same between RNs, RPNs, and PSWs apart from work practices by nursing respondents. Both RPNs and RNs reported the unique skill set used in community nursing and opportunities for learning.

## 5. Discussion

The primary motivation for the study was to determine if there were differences in predictors of ITL among categories of home care workers (RNs, RPNs, and PWSs) to help inform retention and recruitment strategies. While there was no difference in reported ITL among RNs, RPNs, and PSWs, organization commitment was the key predicator of ITL. There were significant differences among some of the predictors, including role stress, work-family conflict, burnout, and one aspect of job-satisfaction with salary and wages. These predictors and differences among categories are discussed .

Overall, the relationships among the study variables were expected. Although weak, higher community satisfaction correlated with lower role stress, lower work-family conflict, and lower burnout. In previous research, community satisfaction was identified as a unique predictor of ITL in rural RNs [3, 24]. To better understand if there was a difference in community satisfaction between rural and urban respondents, additional analysis was completed, yet no difference was found. Therefore, community satisfaction seems to be important regardless of whether one lives or works in urban or rural communities, yet further investigation is warranted, including consideration of how an employer can contribute to community satisfaction or dissatisfaction.

### 5.1. Organizational Commitment

When examining all the predictors of ITL for the entire sample, the most significant predictor of ITL was organizational commitment. The items assessed in the measurement of organization commitment include alignment of one's values with the organizational values. Organizational commitment is a globally a well-recognized predictor of retention in nursing, yet little evidence is available for home care and the different categories of workers. Research that does exist indicates that strategies that promote employee empowerment [27, 28], supervisory support and leadership [27], professional development and training [28], and the implementation of evidence-based leadership approaches [29] can contribute to organizational commitment and thus reduce ITL.

### 5.2. Role Stress

RNs reported higher role stress than both RPNs and PSWs. Upon investigation, the component of role stress that differed was role overload. Role overload is the result of being expected to complete a variety of tasks in insufficient time [18]. The unique skill set of RNs in home care and the concerns noted about scheduling practices may contribute to issues of overload. Workload has been recognized by nurses as contributing to ITL, especially for new nurses, where workload contributes to stress [30]. Additional research could further examine the relationship between workload and category of employment in community care to determine, for example, if and how higher skill set requirements contribute to role stress in the specific environment of community care.

### 5.3. Work-Family Conflict

Both RNs and RPNs reported higher work-family conflict than PSWs. Upon further examination, there were no significant differences in the groups having dependents at home which may contribute to this; however, other contributing factors were not explored to help understand what is contributing to the conflict. From the themes that emerged, traveling and scheduling practice may influence work-family conflict. Studies have shown that work-family conflict is a significant predictor for healthcare workers' ITL and that this is particularly the case for home care workers [31]. However, more research is needed to understand the variables that contribute to this relationship, specifically with respect to different employment categories within home care.

### 5.4. Burnout

RNs reported higher burnout than both RPNs and PSWs. The burnout measure consisted of four subscales, including exhaustion, mental distress, cognitive impact, and emotional impact. Upon further examination, it was the exhaustion subscale that was significantly different. Schaufeli and colleagues [19] describe exhaustion as the “heart of the burnout syndrome” (p. 13) and one of the core symptoms of burnout. The items assessing exhaustion considered both mental and physical exhaustion. Burnout is a recognized predictor of ITL in nurses [9, 32].

These concerns about burnout align with the findings from Moloney and colleagues [9] in New Zealand who reported that workload and work-life interference contributed to burnout in RNs, which in turn was a significant predictor of ITL. Although Moloney's findings were not specific to home care, focused on RNs only, and were in a different jurisdiction, the findings align with this study. The concern of role overload and work-family conflict reported in nursing respondents, especially RNs, are likely contributing to the exhaustion. Therefore, strategies to address workload, work-family conflict, and exhaustion should be considered in a retention strategy.

### 5.5. Dissatisfaction with Salary and Wages

Dissatisfaction with wages and salaries was a quantitative and qualitative finding from the study. Among the categories of work, all three groups reported low satisfaction with salary and wages but there was a significant difference with RPNs, who reported greater dissatisfaction than PSWs. Although details of the salary dissatisfaction were not obtainable, other studies have found that dissatisfaction with wages and compensation greatly contributes to overall job dissatisfaction and ITL [33]. This is especially so when healthcare workers feel there is a disparity between workload and compensation, which is often found to be the case in community care [34]. Dissatisfaction with wages has also been found to contribute to ITL early in employment (e.g., within the first year), particularly for those working in home care [35].

### 5.6. Recommendations

Based on the results of the study, the following recommendations are made. The recommendations support retention.

#### 5.6.1. Address Compensation Dissatisfaction

Addressing the dissatisfaction with compensation includes increasing salary and wages of home care workers and providing additional compensation strategies including mileage, travel time, and benefits are needed. The respondents recognized the differences in wages between healthcare sectors as well as the complexity of seeking wage increases, noting the need to work with the government and advocacy for the change. Although all members of the team can advocate, senior leaders are well positioned and can use existing relationships with government at the provincial level. Advocating for wage parity among healthcare sectors will also signify an understanding and respect for home care. Without this parity, the low wages represent undervalue of home care and the workers in the sector.

Separate from wage parity, other payment strategies can be considered and seen as advantageous for staff. Non-wage-related compensation strategies suggestions include increasing mileage compensation and payment for travel time between clients' homes. For benefits, it was recognized by respondents that the cost of current benefits was disproportionate to their wages. Although the details of the cost of participating in benefit programs were beyond the scope of this study, it is recommended that a review of options and employee costs be undertaken. Many respondents recognized the increased costs associated with the vehicle requirement of working in home care. Specific recommendations to address these include increasing the mileage provided to support the cost of gas and vehicle maintenance. In addition, some respondents suggested offering work that does not require traveling, such as clinics.

#### 5.6.2. Examine Scheduling Practices

A variety of strategies were suggested by respondents for improving scheduling, including some conflicting suggestions (for example, less overtime and more overtime offered). Several strategies can be considered to enhance satisfaction with scheduling in the workplace. It is recommended that practice includes creating a staff scheduling committee and ensuring a common understanding and application of collective agreements. A staffing scheduling committee with staff can help address scheduling concerns raised by staff and serve as a forum for management and supervisors to be present with staff. In addition, revisiting and possibly revising scheduling practices could ensure fairness and efficiency in terms of assignments (less travel time), staff satisfaction, and continuity of clients [3, 36]. In a review of literature on nurse's ITL, Al Zamel et al. [37] highlight the consistent significance of job satisfaction and work environment. They suggest that ensuring job satisfaction includes monitoring and being aware of staff concerns and then implementing specific strategies that address those concerns. Creating a staffing scheduling committee would provide ongoing opportunities to understand and respond to such concerns.

Given the comments regarding fairness in scheduling, it is suggested that where appropriate (i.e., for unionized environments), education to managers, supervisors, and schedulers be provided to help ensure a common understanding of the collective agreements and application, specifically for scheduling. This is consistent with findings in the study conducted by Stewart et al. [3], which examined nurses' intentions to leave in rural care, Canada. Stewart et al. noted that factors contributing to the intention to leave within the next 12 months of the study included high stress and dissatisfaction with scheduling. In contrast, in the study carried out by Tourangeau et al. [8], home care nurses in community settings in Canada reported a higher intention to remain employed when scheduling practices were flexible, work environments were supportive, and workloads were manageable and varied. Opportunities for collaboration, education, and support from leadership are conducive to positive work environments, increased workplace safety, and lower dissatisfaction overall [29, 38]. Managers can take a proactive and open approach to concerns around safety. Butler [38] determined in a study involving 252 home care aides that the ability to discuss concerns with leadership promoted feelings of safety for employees.

#### 5.6.3. Enhanced Management Support and Practices

Managers and supervisors are positioned to promote the team dynamic and demonstrate appreciation and recognition of staff. Based on the findings of this study, the need for recognition for work done and providing support was separate from compensation. On a positive note, the “team” was an important reason for staying with the home care agency. Recommendations to help management (and supervisors) meet identified needs and promote the “team” include creating opportunities for teams to work together, utilizing reward and recognition programs, and implementing a values-based approach to recruitment to promote organizational commitment.

Existing studies support the significance of fostering team cohesion and supportive work environments. In their study involving survey results from 16,707 nurses in England, Carter and Tourangeau [39] conclude that adequate support and resources contribute to a more positive work environment. They further highlight that a positive work environment is a significant factor in employee retention. Nurses who reported feeling more engaged (psychologically involved) in their work had lower rates of ITL. Collegial work settings, improved work relationships, and an emphasis on team building have been shown to benefit home healthcare workers and have been suggested to improve retention [33].

Opportunities for teams to work together, learn about one another, and work directly with managers are needed. If these opportunities are already present, seek feedback from staff about what is missing from the meetings, what can be added to make them more engaging and meaningful, and hear what else might be done. These strategies will not only help open communication channels, create teams, and foster trust among team members, but will also provide an opportunity for management to engage in authentic and transactional leadership practices. There are many resources for supporting team development available, for free, from several organizations.

Additional efforts could be made to implement or strengthen recognition programs. Research shows that perceiving the workplace as fair and feeling appreciated are factors in reducing the intention to leave [29]. If a recognition system already exists, evaluate how it is being used by managers and supervisors, including what barriers might prevent its use and what might encourage its use of the system. Depending on the barriers and staff feedback on the existing system, consider what a new recognition system might look like. There are many examples of innovative recognition systems ranging from formal recognition (e.g., certificate of recognition) to informal types of recognition (e.g., peer-recognition). In addition to being connected with organizational commitment, recognition and rewards in the workplace have been linked to staff resilience and improved mental health [40].

Secondly, given the importance of the link between organizational commitment and retention in the workplace, leaders need strategies that promote organizational commitment as early as the recruitment phase. Organizational commitment has been demonstrated to be a significant factor in ITL in that the higher the organizational commitment, including in healthcare work environments, the lower the level of ITL [5, 29]. In terms of fostering organizational commitment as early as recruitment, one approach to interviewing is values-based [41]. Values-based interviews focus on how and why an individual is oriented towards making certain choices in the workplace. Questions in the values-based approach seek to gain information about what is important to the applicant and assess if their values are appropriate for the role and organization.

#### 5.6.4. Maintain and Expand upon Existing Practices That Support Staff

The findings highlighted several practices that should be reinforced and continued. Respondents were satisfied with orientation, onboarding, and professional development opportunities. These practices are essential in a healthy work environment, which supports recruitment and retention in the workplace. In addition to the connection with clients/patients, the “love” and “joy” staff reported to have received from providing care were evident and an overwhelming finding from the comments received.

Ongoing monitoring of the onboarding and orientation activities, inclusive of feedback from participants should be practiced to continually improve in these areas. In their literature review of research on job satisfaction, [42] note that “what makes a job satisfying or dissatisfying does not only depend on the nature of the job but also on the expectations that individuals have of what their job should provide” (p. 1018). Many of the studies examining ITL in healthcare incorporate self-report surveys, and employees consistently report that support from managers as well as opportunities for engagement and professional development contribute to job satisfaction. Professional development opportunities for all staff could be enhanced with special consideration for nursing and the unique skill set required by nursing in home and community care. An approach that takes into consideration the specific circumstances of home and community care is more likely to encourage retention in this sector [34].

Leaders can explore opportunities to learn more about what can be done to help employees find joy in home care and/or remove barriers so that they have improved work enjoyment. The Institute of Health Improvement, Framework for Improving Joy in Work, is a free resource that organizations can use to help guide leaders identify barriers to joy in the workplace and work with colleagues to create meaningful strategies. This suggestion is consistent with literature demonstrating that healthcare providers who feel more engaged in their workplace, who have a sense of being part of a team, and who are provided opportunities for being “psychologically involved” in their work report lower ITL [39]. Seeking opportunities for a more positive work environment that emphasizes teamwork can contribute to job satisfaction and subsequent retention [43].

### 5.7. Limitations

There are several limitations to this study, including the use of a convenience sample, the cross-sectional design method, self-reporting, and the sample size. Future studies would benefit from a random sample, with multiple employers and additional measures. The self-reporting approach to data collection used in this study has the potential for response bias such as carelessness, social desirability, or acquiescence [44]. Furthermore, the study and analysis required a high response rate. Although there was good representation across each category of worker, more responses were desired from RNs and RPNs to help distinguish differences among the groups of workers. Therefore, generalization of the results is limited, and future research is needed with consideration for other research methods and larger sample sizes.

In addition, the survey was circulated and completed during the COVID-19 pandemic. Although limited comments were received related to the pandemic in the open-ended items, the impact of the pandemic on healthcare providers must be acknowledged as a potential limitation to the study. Results may be influenced by the ongoing stress of being an essential service, including but not limited to working short-staffed due to restrictions on multiple employers imposed by the government and the risk of transmissibility in the community.

## 6. Conclusion

This study has contributed to the existing literature by examining the unique factors that influence ITL among home care providers in three categories: registered practical nurses, registered nurses, and personal support workers. In assessing and comparing predictors of ITL (organizational commitment, job satisfaction, perceived supervisor support, burnout, role stress, work/family conflict, and community satisfaction) for 185 respondents, this study has led to several significant implications for possibilities to improve both recruitment and retention in home care.

The chief concern among all three categories of care providers was dissatisfaction with wages and salaries. Strategies aimed at wage parity across the healthcare sector and increased opportunities for compensation for home care workers should be encouraged to improve recruitment and retention. Another commonality among the categories was an expressed dedication to the clients, to the team, and to the job. However, inadequate pay and insufficient recognition of the unique circumstances of home care contributed to feelings of being underappreciated and dissatisfied. As such, scheduling practices should be revisited and improved to ensure fairness and respond to the specific concerns and needs of home care providers. Incorporating or enhancing opportunities for team cohesion, management and supervisor support, and staff recognition are also suggested for both recruitment and retention efforts. The implications of high rates of turnover in healthcare represent an ongoing concern and a growing issue, particularly in the face of an aging population and the lingering impacts of a global pandemic. This paper contributes to an understanding of ITL in home care and offers suggestions to improve recruitment and retention specific to the unique work environment of home care.

## Figures and Tables

**Table 1:** Measurement tools assessing key constructs of conceptual framework.

Component of theoretical model	Variable	Measurement tool (source)
Personal factors	Home-life obligations	Work-family conflict [17]
	Stress	Role stress [18]
	Burnout	Burnout assessment tool [19]

Job satisfaction	Job satisfaction	Global job satisfaction [20]
	Aspects of job	Component of Casey-Fink new nurse experience survey [21]

Workplace organization	Organizational commitment	Organizational commitment questionnaire [22]
	Supervisor support	Perceived supervisor support scale [23]

Community characteristics and satisfaction	Community satisfaction	Community satisfaction [24]

ITL	Intention to leave	Turnover intention [25]

**Table 2:** Characteristics of study participants (*n* = 185).

Characteristic	Frequency (*n*)	Percentage	Mean (SD)
*Role description*			
RN	46	25	
RPN	46	25	
PSW	77	42	
Length of time working in healthcare (years)			15.65 (11.72)
Length of time employed with the agency (years)			8.14 (8.87)
*Number of jobs (including with the agency)*			
One	115	62	
Two	33	18	
Three	3	2	
*Job status with the agency*			
Full-time	109	59	
Part-time	44	24	
Casual	17	9	
*Sex*			
Female	150	81	
Male	14	8	
Age (years)			46.1 (12.48)
*Marital status*			
Single	52	28	
Married	102	55	
Other (included widowed, divorced or separated)	14	8	
*Dependents*			
Yes	106	57	
No	62	34	
*Size of community*			
Metropolitan	56	30	
Community	82	44	
Rural	34	18	
*Education level*			
High school	5	3	
College certificate	36	20	
College diploma	80	43	
University undergraduate degree	29	16	
University graduate degree	17	9	

**Table 3:** Study measurement of variables (ranges, means and reliability coefficients).

Scale (subscales)	Range	Mean (SD)	Reliability coefficients (Cronbach's alpha)
Organizational commitment	9–63	39.72 (12.31)	0.91
Role stress	8–40	21.55 (6.85)	0.84
Role ambiguity	5–25	12.35 (4.07)	0.75
Role overload	3–15	9.21 (3.46)	0.80
Work family conflict	5–25	15.39 (5.90)	0.93
Job satisfaction	10–50	33.73 (8.37)	0.87
Turnover intention	4–20	11.06 (5.13)	0.93
Community satisfaction	11–55	49.63 (7.70)	0.88
Perceived supervisor support	16–112	72.24 (24.67)	0.97
Burnout assessment tool	23–115	51.29 (14.46)	0.93
Exhaustion	8–40	23.79 (7.10)	0.91
Mental distance	5–25	10.91 (4.09)	0.81
Cognitive impact	5–25	8.09 (3.42)	0.89
Emotional impact	5–25	7.83 (2.95)	0.81

**Table 4:** Pearson's correlations among study variables.

	Organizational commitment	Role stress	Work-family conflict	Job satisfaction	Supervisor support	Turnover intention	Community satisfaction
Role stress	−0.420^*∗∗*^						
Work-family conflict	−0.285^*∗∗*^	0.505^*∗∗*^					
Job satisfaction	0.709^*∗∗*^	−0.483^*∗∗*^	−0.378^*∗∗*^				
Supervisor support	0.518^*∗∗*^	−0.424^*∗∗*^	−0.340^*∗∗*^	0.742^*∗∗*^			
Turnover intention	−0.575^*∗∗*^	0.419^*∗∗*^	0.359^*∗∗*^	−0.584^*∗∗*^	−0.420^*∗∗*^		
Community satisfaction	0.269^*∗∗*^	−0.167^*∗*^	−0.176^*∗*^	0.285^*∗∗*^	0.184^*∗*^	−0.188^*∗*^	
Burnout	−0.392^*∗∗*^	0.601^*∗∗*^	0.544^*∗∗*^	−0.437^*∗∗*^	−0.438^*∗∗*^	0.377^*∗∗*^	−0.198^*∗*^

^*∗∗*^Correlation is significant at the 0.01 level (2-tailed). ^*∗*^Correlation is significant at the 0.05 level (2-tailed).

**Table 5:** Results of one-way analysis of variance.

Measure	Mean by group (standard deviation)			*F* value	*p* value
	RNs	RPNs	PSWs		
Organizational commitment	39.52 (10.30)	38.74 (12.41)	41.32 (12.89)	0.68	0.51
Role stress	23.80 (6.82)	22.05 (6.73)	19.55 (6.54)	5.69	<0.05
Work family conflict	17.16 (5.74)	16.68 (5.79)	14.00 (5.45)	5.57	<0.05
Job satisfaction	33.80 (7.78)	33.33 (7.58)	34.30 (8.72)	0.19	0.83
Supervisor support	73.19 (24.74)	73.86 (26.42)	73.55 (22.78)	0.01	0.99
Turnover intentions	11.40 (5.12)	11.67 (5.19)	10.14 (4.90)	1.61	0.20
Community satisfaction	49.12 (7.73)	50.88 (7.07)	48.90 (8.01)	0.94	0.39
Burnout	54.77 (14.02)	51.85 (13.55)	48.18 (14.00)	3.02	0.05
Satisfaction with salary	2.11 (1.30)	1.59 (0.70)	2.26 (1.31)	7.76	<0.001

**Table 6:** Themes on strategies to keep staff.

Theme	Details from respondents	Quotes
Compensation	Overwhelming theme across the three groups, with suggestions for salary and wage increases Discrepancies between sectors acknowledged, and not reflective of responsibilities Additional types of compensation acknowledged (improved vacation, benefits, bonuses for “staying,” improved mileage or provision of gas cards)	“Pay parity with hospitals”

Scheduling	Desire for greater continuity of patient assignment Fewer weekends Less pressure to pick up shifts Job sharing opportunities Improve scheduling to reduce driving Options for settings that don't require driving (e.g., clinics) Avoid consecutive days or shifts More staff needed to improve workloads and scheduling Provide time for hand-off Refrain from mandatory overtime	“Almost every shift” (mandatory overtime)

Management opportunities	Treat staff fairly and provide recognition for their work Personal contact-listening, showing concern (including for mental health) Improved communication and approachability more training and education^*∗*^ Address concerns of safety and accommodations^*∗∗*^	“Favouritism” “It's not fair” “Overall, not appreciated”

^*∗*^RN and RPN respondents. ^*∗∗*^RPN respondents.

**Table 7:** Themes related to why employees stay.

Theme	Details from respondents	Quotes
Patient/clients	“Love” for clients Concern for clients if not there	“Wake up every morning excited to go to work,” “the patients keep me…”

Nature of community care	Being close to home Having an impact on the community The duties work and skills (critical thinking)^*∗*^	“Believe in the value, necessity and quality of community health care”

The “team”	The company, management, and coworkers Friendly and helpful coworkers	“Feels like a family,” “amazing supervisor” “Love the team”

Work practices	Having vacation and sick days, flexibility and independence, availability of overtime Opportunities for continued learning	“Love the support”

Financial need	Years of services and pension stopped employees from leaving Need for job/wages contractual obligation	

^*∗*^RN and RPN respondents.

## Data Availability

The data used to support the findings of this study are restricted. Participants of this study did not give written consent for their data to be shared publicly.

## Additional Points

*What Is Known about This Topic?* (i) ITL is known to be a key predictor of turnover, influenced by organizational, personal, and community factors. (ii) The models of ITL have been studied on Registered Nurses but limited evidence is available assessing models with different workers. (iii) Home care faces unique challenges which may influence ITL and retention. *What This Paper Adds?* (i) Similarities and differences of predictors of ITL among Registered Nurses, Registered Practical Nurses, and Personal Support Workers working in home care are reported. (ii) Dissatisfaction with wages and salary was reported by the three categories, along with recognition of disparity across sectors. For some respondents, wage disparity contributes to the feeling that home care is undervalued and underappreciated. (iii) Unique considerations for home care workers related to retention include scheduling, continuity of clients assigned, travel time, concerns of mileage and related expenses, and safety with traveling and being in the community.
